# Malnutrition-related conditions and interventions in US state/territorial Older Americans Act aging plans

**DOI:** 10.1186/s12877-022-03342-7

**Published:** 2022-08-13

**Authors:** Mary Beth Arensberg, Jaime J. Gahche, Johanna T. Dwyer, Adam Mosey, Damon Terzaghi

**Affiliations:** 1 Health Policy and Programs, Abbott Nutrition Division of Abbott, 3300 Stelzer Road, Columbus, OH 43219 USA; 2 Office of Dietary Supplements, National Institutes of Health, 6705 Rockledge Dr, Bethesda, MD 20817 USA; 3 Frances Stern Nutrition Center at Tufts Medical Center, Boston, MA USA; 4 Departments of Medicine and Community Health Tufts University School of Medicine and Gerald J. and Dorothy R. Friedman School of Nutrition Science and Policy, Tufts University, Boston, MA USA; 5 The Jean Mayer United States Department of Agriculture Human Nutrition Research Center On Aging at Tufts University, Boston, MA USA; 6 Aging Policy, ADvancing States, 241 18th Street S, Suite 403, Arlington, VA 22202 USA; 7 LTSS Policy, ADvancing States, 241 18th Street S, Suite 403, Arlington, VA 22202 USA

**Keywords:** Older Americans Act (OAA) aging plans, Malnutrition, Sarcopenia, Frailty, Obesity, Older adults, Nutrition

## Abstract

**Background:**

Factors that decrease independence and increase morbidity must be reduced to improve the nutrition, health, and other challenges confronting older adults. In the United States (US), the Older Americans Act (OAA) requires each state/territory develop multi-year aging plans for spending federal funds that foster healthy aging (including support of congregate/home delivered meals programs) and separately requires grant applications for nutrition service programs supporting older Native Americans. Malnutrition (particularly protein-energy undernutrition), sarcopenia, frailty, and obesity can all result in disability but are potentially changeable. The study goal was to collect baseline information on mentions of these malnutrition-related conditions and interventions that address them in US state/territorial OAA program multi-year aging plans.

**Methods:**

OAA program multi-year aging plans available on the ADvancing States website in February 2021 (*n* = 52) were searched for number of mentions of defined nutrition terms including malnutrition, sarcopenia, frailty, obesity, and whether terms were included in plans’ goals/objectives, strategies/actions, or solely in the narrative.

**Results:**

Malnutrition, sarcopenia, frailty, and obesity were mentioned infrequently in US state/territorial OAA program multi-year aging plans. 33% of plans mentioned malnutrition but only 8% as goals/objectives and 15% as strategies/actions. 62% mentioned frailty; 6% (goals/objectives), 15% (strategies/actions). None mentioned sarcopenia whereas in contrast, 21% mentioned obesity; 2% (goals/objectives), 2% (strategies/actions). Nutrition intervention mentions were nearly nil. There were no significant differences in frequency of term mentions by US region or by states with higher percentages of older adults or obese adults.

**Conclusions:**

Clearly specifying definitions of malnutrition-related conditions and incorporating them into measurable goals/objectives, defined strategies/actions, and outcomes may help improve future state/territorial OAA program multi-year aging plans to better support healthy aging.

## Background

In many countries, policymakers and public health providers’ increasing recognition of the importance of nutrition and other social determinants of health as well as powerful economic incentives have led them to help older adults successfully age in place by moving more services from clinical to community settings [1]. Several nutrition-related conditions (malnutrition, sarcopenia, frailty, and obesity) are partially preventable conditions that are all included in the World Health Organization (WHO)’s care pathway for managing malnutrition [2]. Yet when unidentified and untreated these conditions negatively affect community-dwelling older adults’ quality of life, social, functional, and health outcomes [3–5].

Prevalence rates of these conditions among older adults in the community can vary. The prevalence of overweight and obesity tends to be well-documented, including in the United States (US). However, data on the national prevalence of malnutrition, sarcopenia, and frailty among community-living older Americans is lacking, although the prevalence is thought to be substantial [6]. The joint action Malnutrition in the Elderly (MaNuEL) Knowledge Hub has documented the prevalence of protein-energy malnutrition/undernutrition (referred to as malnutrition in this article) among 7 countries (Austria, France, Germany, Ireland, Spain, The Netherlands, and New Zealand) [7] and potentially provides a model for similar documentation efforts in the US. A recent systematic review estimated the prevalence of malnutrition among community living older adults in North America to be 6.1% [8]. Internationally, sarcopenia prevalence estimates are 1–29% for community-dwelling older adults, depending on definitions used and locale [9]. Sarcopenia prevalence rates are not clearly defined in the US. Globally the community prevalence of frailty in older adults varies from 4–59%, but frailty rates are not tracked in the US [10].

Documentation of these conditions is critical in the US, because the managers of government programs for older adults are increasingly confronted with larger numbers of very old individuals with multiple chronic conditions and disabilities. For example, by the end of the decade 20% of the US population will be 65 years or older, with those aged 85 + years representing the fastest growing age group [1]. Many older adults are afflicted with age-related conditions resulting in difficulties in carrying out activities of daily living [11]. To meet these challenges and improve public health, it is imperative to focus on prevention and reduce potentially modifiable conditions such as malnutrition, sarcopenia, frailty, and obesity, that increase morbidity and decrease independence [12–15].

The primary US community-based, federal nutrition programs that support older adults to help them age in place, regardless of income, are those funded by the US Older Americans Act (OAA) [16]. These include congregate/home-delivered nutrition programs and nutrition services for older Native Americans (OAA Title III and VI programs, respectively). In 2020, the US Congress required inclusion of reducing malnutrition in the OAA Title III Nutrition Services Program’s purpose and the addition of malnutrition screening to the Program’s disease prevention/health promotion services definitions [17].

The American dilemma is that there are several challenges in implementing and monitoring such national mandates to improve the nutrition of older persons at the community level. First, the prevalence of some malnutrition-related conditions is unknown nationally. State/local administrators can differ in their views about what these conditions are and how to best deal with them. Also, the OAA nutrition programs must be implemented within a highly decentralized, fragmented health care and social services system and an OAA infrastructure designed a half-century ago for what was then a much smaller, younger, and healthier older adult population.

A logical step toward helping resolve this dilemma is regular evaluation of the multi-year aging plans US states/territories are required to develop for OAA programs. However, initial information on current nutrition-related content and objectives of each state and related entity’s multi-year plan is lacking. This study documents a first approach to obtaining baseline information on whether US state/territorial OAA program multi-year aging plans mention specific nutrition-related conditions (malnutrition, sarcopenia, frailty, and obesity) and interventions that could positively impact healthy aging. The results may provide insights for those in other countries encountering similar challenges in translating national guidance into local actions.

## Methods

### Study sample

The study sample comprised all publicly available US state/territorial OAA program multi-year aging plans. In February 2021, 52 plans (50 states, Washington DC, and Guam) were available and accessible through the ADvancing States website, which provides a map directly linking to individual plans [18]. ADvancing States represents 56 US state/territorial units on aging and disabilities and long-term services/supports directors whose responsibilities include implementing the federally funded OAA nutrition programs.

### Measurement

Our baseline information study sought to quantify frequency of mentions in US state/territorial OAA program multi-year aging plans, and related federal guidance documents, of specific nutrition-related conditions (malnutrition, sarcopenia, frailty, and obesity) and interventions that could impact healthy aging. The goal was to quantify both the overall number of mentions as well as the number of mentions in any goals/objectives and/or strategies/actions.

First, 3 researchers defined nutrition terms that would be searched in the individual state/territorial plans. These defined nutrition terms were nutrition, malnutrition/underweight/undernutrition, sarcopenia, frail/frailty, obesity/overweight, and dietary supplements/oral nutrition supplements/meal replacements. Both sarcopenia and frailty were included because it was believed that many policymakers may not distinguish between the two. Further, while the specific number of terms was limited, the focus was on conditions/interventions related to malnutrition, muscle mass, body composition, and functionality, thus other chronic diseases or clinical conditions with nutrition implications, such as diabetes or swallowing disorders were not included in the search terms.

The same 3 investigators next conducted an initial review of 5 state/territorial plans to develop the search process for the defined nutrition terms within the plan documents. Specifically, after they independently searched the same 5 plans, results were examined, any differences were adjudicated, and a consistent search approach was confirmed. The investigators used a simple electronic word find function to search each plan document, recording the overall numeric frequency of mentions of the defined nutrition terms as well as frequency of mentions in any goals/objectives and/or strategies/actions. Final analyses were completed by April 2021; the 3 investigators independently reviewed 1/3^rd^ of alphabetically ordered remaining 47 plans, with intentional overlap (2 investigators reviewing the same plan) for 19% of plans.

As the US Administration on Community Living (ACL) administers OAA programs, in February 2021, the 3 investigators also searched ACL’s guidance at that time for state/territorial OAA program multi-year aging plans [19] and for OAA Title VI grants supporting older Native Americans [20, 21] for mentions of the defined nutrition terms. The individual OAA Title VI grant applications were not publicly available and could not be searched.

### Statistical analysis

Statistical analyses were conducted using SAS 9.4 (SAS Institute, Inc, Cary, NC). The percentage of states/territories mentioning at least one of the defined terms was calculated. Means, standard deviations and quartiles were estimated for each of the defined terms. Finally, the mean number of mentions was compared among states with lower and higher percentages of older adults and obesity in their population. “Lower” and “higher” were defined by the lower 50^th^ percentile compared to the highest percentile of older adults or obese adults as a percentage of the state’s population. State estimates of obesity were obtained from the US Centers for Disease Control and Prevention’s Nutrition, Physical Activity, and Obesity: Data, Trends and Maps [22]. Differences between groups were tested using generalized linear models to calculate the F statistic and all significant differences reported were significant at the *p* < 0.05 significance level.

## Results

**Fig. 1:**
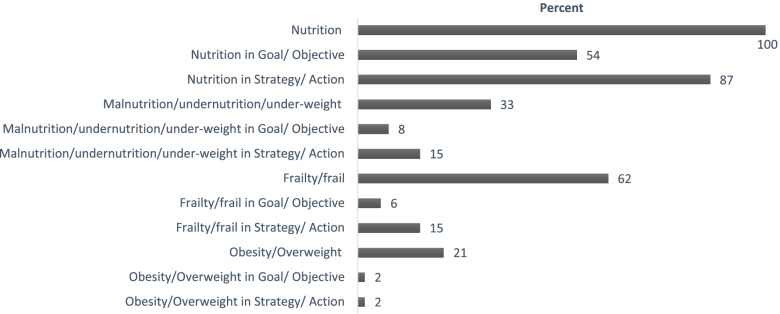
Percentage of 52 state/territory multi-year aging plans with at least one mention of defined nutrition terms* ^*^ US state/territorial unit on aging multi-year aging plans reviewed were those available on the ADvancing States website in February 2021; results reflect percentage of at least one mention in overall plan and in plan goal/objective, and strategy/action

US state/territorial OAA program multi-year aging plans were available for 50 states, the District of Columbia, and 1 US territory (Guam) on the ADvancing States website in February 2021. The term *nutrition* was included at least once in all 52 state/territorial aging plans (Fig. 1). About 54% of states/territories mentioned it once or more as part of goals/objectives and 87% included it under strategies/actions. In contrast, specific mentions of the terms related to body composition, muscle mass, and functionality were far fewer. Of the 52 state/territorial plans reviewed, 33% mentioned *malnutrition/undernutrition/underweight* at least once; but only 8% of mentions were as part of goals/objectives and 15% as part of strategies/actions. The terms *frailty/frail* were mentioned in 62% of the plans; however, only 6% of mentions were in goals/objectives and 15% in strategies/actions. Furthermore, 21% of the plans mentioned *obesity/overweight* and only 2% mentioned in goals/objectives, and 2% in strategies/actions. There was little mention of interventions *dietary supplements/oral nutrition supplements/dietary supplements* and no plans mentioned *sarcopenia*.

Overall, in state/territorial plans the mean number of mentions of *nutrition* was relatively high; 31.1 ± 23.0 SD, although the number varied greatly from one state to another (Table 1). Fifty percent of plans had 25.5 or more mentions of *nutrition* and 25% of plans had 40.5 or more mentions of *nutrition*. However, most mentions did not single out *nutrition* in goals/objectives (mean 1.8 ± 3.3) or strategies/actions (mean 6.6 ± 10.1), rather they simply referred to *nutrition* in the verbiage describing more general aspects of the aging plan and its programs. In contrast, none of the terms related to body composition, muscle mass, and functionality such as *malnutrition/undernutrition/underweight, sarcopenia, obesity/overweight* had a mean that rounded to even a single mention. Frailty was mentioned on average 2.7 times in aging plans but only in the text, and on average not in goals/objectives or in strategies/actions.

**Table 1:** Descriptive statistics for defined nutrition term mentions in 52 US state/territorial multi-year aging plans^a^

Terms searched	Mean	Standard deviation	25th quartile	Median	75th quartile
Nutrition					
Number (#) overall mentions	31.1	23.0	14.5	25.5	40.5
# mentions in Goal/Objective	1.8	3.3	0.0	1.0	2.0
# mentions in Strategy/Action	6.6	10.1	1.0	4.0	7.0
Malnutrition/undernutrition/underweight					
# overall mentions	1.2	3.2	0.0	0.0	1.0
# mentions in Goal/Objective	0.1	0.4	0.0	0.0	0.0
# mentions in Strategy/Action	0.3	1.1	0.0	0.0	0.0
Frailty/frail					
# overall mentions	2.7	4.0	0.0	1.0	3.5
# mentions in Goal/Objective	0.1	0.3	0.0	0.0	0.0
# mentions in Strategy/Action	0.2	0.5	0.0	0.0	0.0
Obesity/overweight					
# overall mentions	0.4	1.0	0.0	0.0	0.0
# mentions in Goal/Objective	0.0	0.1	0.0	0.0	0.0
# mentions in Strategy/Action	0.1	0.4	0.0	0.0	0.0

^a^Plans reviewed were those available on the ADvancing States website in February 2021; there were no mentions of sarcopenia in the state/territorial unit on aging plans

To test whether US states/territories with a large proportion of older persons might give more attention to nutrition, the mean number of mentions for the defined nutrition terms was compared between states with the lowest and highest percentage of older adults in the state’s population. *Nutrition* was mentioned on average 27.4 times among states with a lower percentage of older adults compared to 35.2 times among states with a higher percentage of older adults, but the difference was not statistically different (Table 2). Similarly, no significant differences were found between states with lower compared to higher percentages of older adults for any of the other defined nutrition terms. No statistically significant differences were evident either between the mean number of mentions for the defined nutrition terms when states that had lower percentages of obesity were compared with those with higher obesity (data not shown).

**Table 2:** Mean number defined nutrition term mentions in state multi-year aging plans by percentile US state population ≥ 65^a^

Terms searched	Percent (%) of the population that is ≥ 65 years of age^b^	
	< 15.5%	> 15.5%
Nutrition		
Number (#) overall mentions	27.4	35.2
# mentions in Goal/Objective	1.7	1.9
# mentions in Strategy/Action	5.9	7.4
Malnutrition/undernutrition/underweight		
# overall mentions	1.1	1.2
# mentions in Goal/Objective	0.1	0.2
# mentions in Strategy/Action	0.4	0.3
Frailty/frail		
# overall mentions	2.6	2.8
# mentions in Goal/Objective	0.1	0.0
# mentions in Strategy/Action	0.3	0.1
Obesity/overweight		
# overall mentions	0.5	0.4
# mentions in Goal/Objective)	0.0	0.0
# mentions in Strategy/Action)	0.1	0.0

^a^Mentions of defined nutrition terms in 50 US state unit on multi-year aging plans by those available on the ADvancing States website in February 2021 and grouped by lower, upper 50^th^ percentile of US state population ≥ 65 years of age; there were no mentions of sarcopenia in the state unit on aging plans

^b^Statistical differences were not found for any terms by % of the population that was ≥ 65 years of age

**Fig. 2:**
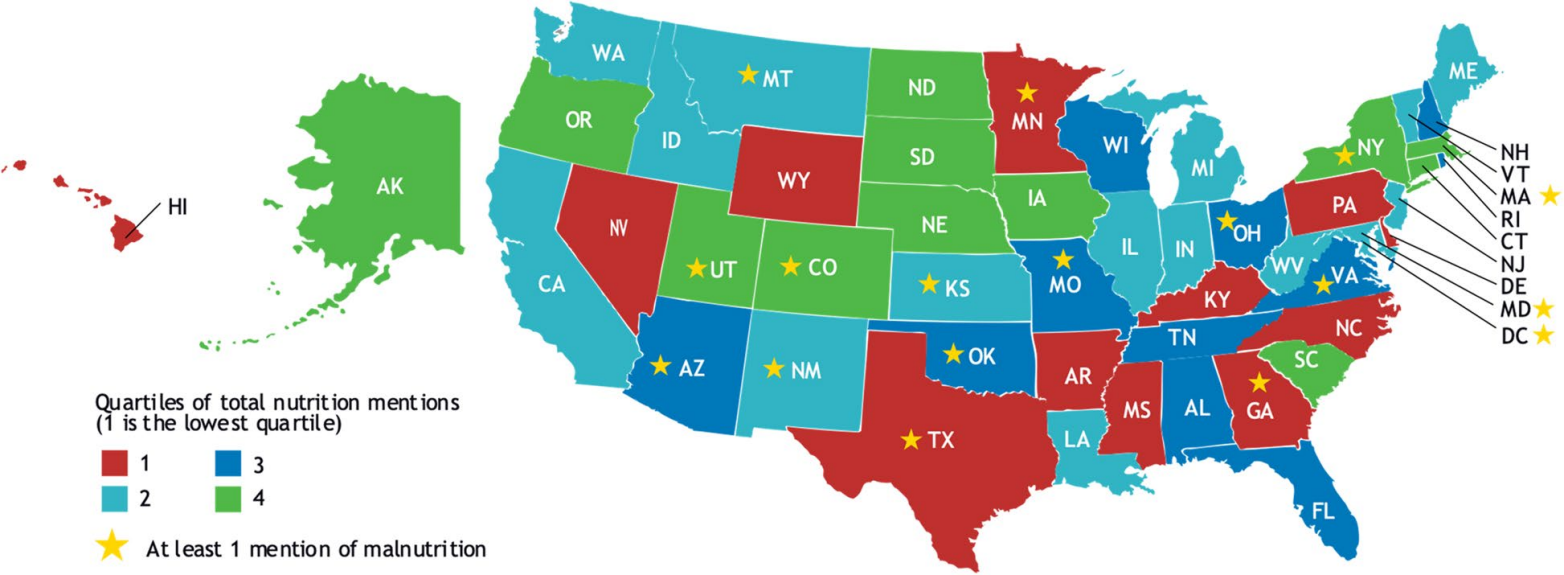
US states by quartile of nutrition mentions and higher number malnutrition mentions in state multi-year aging plans* *State unit on aging multi-year plans reviewed were those available on the ADvancing States website in February 2021. Source: adapted with permission, ©stringerphotography/123RF.COM

Figure 2 depicts the term *nutrition* by quartile of mentions. It also indicates the US states/territories that had a higher number of mentions of *malnutrition/undernutrition/underweight* anywhere in their plans. Higher mentions were defined as ≥ 1 mention compared to 0 mentions for the states as a whole. Only Colorado, Massachusetts, New York, and Utah stood out; their plans fell in the top quartile of mentions of *nutrition* among all the states and had higher mentions of *malnutrition/undernutrition/underweight*. No significant differences in the number of mentions or associations were observed for states with higher number of mentions of *nutrition* and *malnutrition/undernutrition/underweight* singly or both terms together, or when evaluated by states grouped regionally.

In summary, while nutrition was frequently mentioned in state/territorial plans, it was difficult to discern whether the term referred to a program description, the type/amount of food provided by OAA programs, or to the participants’ nutrition status. Nutrition was rarely defined operationally and mentioned infrequently in the goals/objectives and strategies/actions of the plans. There were also very few mentions and only rare inclusions in goals/objectives or strategies/actions of the other nutrition-related terms searched, and it was not possible to determine if they referred specifically to nutrition-related conditions or to possible nutrition interventions.

In the US federal ACL guidance available in February 2021 (for either the state/territorial plans or the Title VI grants supporting older Native Americans) there were no mentions of *malnutrition*, *sarcopenia*, or *obesity*; *frailty* was mentioned only once in ACL’s OAA Title VI guidance.

## Discussion

**Fig. 3:**
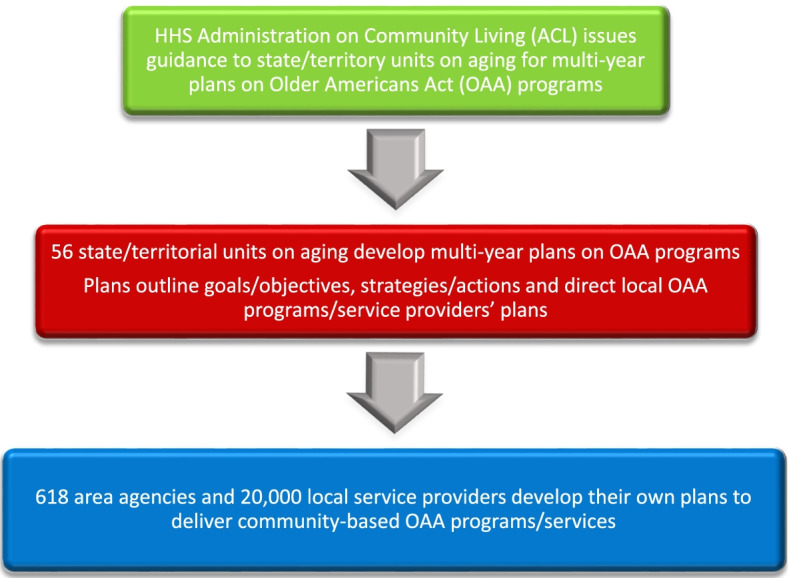
Cascade of US Older Americans Act (OAA) program state/territorial multi-year aging plans

This study provides baseline information on nutrition-related terms described in US state/territorial OAA program multi-year aging plans that was unavailable in any other studies in the literature. The findings underscore a lack of attention to nutrition-related conditions in the plans. There are likely multiple factors at the federal, state, and local levels contributing to this and specific policy responses could lead to improvements at each of these levels. However, this discussion focuses primarily on opportunities at the federal level, since OAA nutrition programs are federally funded. Further, state/territories are required to respond to federal directives and the state/territorial plans in turn serve as the basis for local agency planning (Fig. 3).

### More explicit federal operational definitions, training, and guidance

The baseline finding of limited nutrition-related terms mentioned in state/territorial plans underscores the lack of attention to nutrition-related conditions in them. Policy development is one of 3 core tenants of public health [23]. The absence of a clear operational definition for nutrition in federal guidance may have led to confusion at the state/territorial level and a failure to more frequently include nutrition in state/territorial plans’ goals/objectives and strategies/actions. The provision of operational definitions in future federal guidance for all nutrition-related terms, along with potential strategies and actions associated with these terms would be helpful. Operational definitions are also needed to provide uniform descriptions and definitions for the various ways nutrition screening and intervention contribute to OAA program goals of maintaining/improving the nutrition status of program participants and serving high quality, safe, and healthy food to meet their nutrition needs and health outcomes.

It may be that the limited mentions of nutrition-related conditions and interventions in state/territorial plans reflected limited oversight by registered dietitian nutritionists (RDNs) at state/territorial levels. OAA programs encompass a range of important social services administered by social service personnel and without a requirement that RDNs administer state/territory OAA nutrition programs, greater emphasis may be placed on non-nutrition programs in the OAA program plans. The 2020 OAA reauthorization specified an RDN must federally administer OAA nutrition programs at the federal level [17] and this may lead to stronger nutrition-specific guidance in the future for state/territorial OAA nutrition programs. States/territories could adopt a similar approach by requiring an RDN administer state/territorial OAA nutrition programs to complement the social service expertise already present with oversight for other OAA and social/environmental determinants of the health programs.

It is gratifying to learn that since our study was completed, some steps are being taken toward more explicit and strengthened federal guidance on nutrition and malnutrition. At the time the state/territorial plans that we reviewed were formulated, available federal guidance did not explicitly address malnutrition, sarcopenia, frailty, or obesity; all common conditions among older adults [11]. In its fall 2021 federal training on development of state/territorial OAA program multi-year aging plans, the ACL for the first time included reducing malnutrition, advancing equity, and expanding access to home and community-based services [24]. The ACL guidance for plans that become effective on/after October 2022, requires states/territories address malnutrition in their future nutrition programming, specifically that those plans’ objectives and measures must demonstrate progress toward the goal of “ensuring incorporation of the new purpose of nutrition programming to include addressing malnutrition” [25].

However, much remains to be done. No new federal guidance on OAA grants for older Native Americans (Title VI) has been identified, even though there is very strong evidence of poorer health and nutrition status among this segment. Native Americans represent less than 2% of the US population, but nationally they have some of the highest rates of food insecurity, poverty, diet-related diseases, and other socioeconomic challenges of any group in the US [26, 27]. Malnutrition, frailty, and the social determinants of health are areas of particular concern among Native Americans [28, 29]. Native American tribes have a unique legal relationship as sovereign governments within the US and their members are entitled to special rights, including free healthcare. This reality provides an area for future development and collaboration at the federal level between the US ACL, the US Bureau of Indian Affairs, the Indian Health Service, and other federal agencies engaged in nutrition and health services with a focus on health disparities. It also offers the opportunity to jointly produce updated federal guidance for OAA Title VI programs that addresses malnutrition, sarcopenia, frailty, and obesity and is specifically tailored to this extremely disadvantaged group.

Further, OAA nutrition programs are required by federal law to follow the recommendations of the US Dietary Guidelines for Americans (DGA) which in its most recent edition specifically identified obesity as a concern for all adults and malnutrition, sarcopenia, and frailty as concerns for older adults [30]. It is still too early to know how the latest DGAs and new directives from the ACL may influence future state/territorial OAA program multi-year aging plans, but changes in these plans to target nutrition-related conditions are important to better support healthy aging.

### Larger issues needing attention

The OAA congregate meals programs provide community meals which enhance social interactions and the OAA home delivered meals programs are targeted to older infirm individuals. Neither of these programs can be expected to address all the social and health-related determinants of nutrition. Larger issues related to the entire health and social services system are involved that must also be considered, including those related to the other 2 core tenants of public health: assessment and assurance [23]. Specifically, more complete prevalence estimates and strengthening ties to other community health programs are needed.

#### More complete prevalence estimates for nutrition-related conditions

The paucity of information on prevalence of malnutrition, sarcopenia, and frailty nationally as well as among specific older adult communities and OAA program participant populations may have partly accounted for the low number of mentions of these conditions in state/territorial OAA program multi-year aging plans. State OAA agencies may have assumed that all participants were at risk and there was no need for further focus since many older adults exhibit at least one nutrition-related condition. However, public health data is fundamental to the ability to study conditions within a population [31]. Better data on the prevalence and overlap of malnutrition, sarcopenia, and frailty, as well as obesity among OAA participants is essential because interventions to prevent/treat these conditions vary greatly depending on underlying causes [6]. More complete prevalence estimates on nutrition related conditions can help to target and expand access to home and community-based services. ACL took a positive step forward in convening a technical expert panel in early 2022 to make recommendations for improving its measurement of older adult food insecurity and malnutrition in the ACL National Survey of Older Americans Act Participants [32]. Such data could be used by the ACL’s National Resource Center on Nutrition and Aging to lay the groundwork for prevalence/outcomes research on nutrition-related conditions.

US states where greater attention to older adult nutrition should be of particular concern are California, Florida, and Texas (since 25% of all older Americans will reside in these states by decade’s end) and Georgia, Illinois, Michigan, New York, North Carolina, Ohio, and Pennsylvania (which will account for another 25% of the older American population) [1]. Yet in our study, only New York was among the states whose OAA program multi-year aging plan was in the top quartile of mentions of *nutrition* and had higher mentions of *malnutrition/undernutrition/underweight.*

Failure to screen and to direct appropriate interventions to OAA participants who are most likely to respond may dilute program effectiveness. Estimates of the prevalence of malnutrition, sarcopenia, frailty, and obesity among older adults, using standard, well-validated screening and assessment measures are needed for planning effective interventions. Both malnutrition and obesity were among the chronic conditions identified in a recent report on the effects of OAA participation on health care utilization, but there was no analysis specific to OAA programs and malnutrition and obesity outcomes; in fact, sarcopenia and frailty were not even identified [33]. Future federal guidance with standard definitions could provide items to be used within programs for screening for malnutrition, sarcopenia, frailty, and obesity. Documentation of the prevalence of these conditions, provision of technical assistance, and additional resources for interventions are vital in an OAA aging services network that is already stretched thin [34].

#### Stronger links with other programs/resources impacting nutrition and health

A multiplicity of factors have an impact on the nutrition of older adults [35, 36]. Health disparities across racial and ethnic groups clearly contribute to the burden of malnutrition in the US and are major foci of interest within both the legislative and executive branches of government [37–39]. OAA nutrition programs target older adults in greatest social and economic need, with particular attention to low-income and minority older adults among other groups [40]. Our findings suggest there is a need for increased awareness about the issues of malnutrition and other related conditions and interventions which could impact health disparities in OAA nutrition programs for older adults.

Our study documented no statistically significant associations between number of terms mentioned, percent of state populations who were aged 65 + , percent of state populations who were obese, or states grouped by region of the country. The findings suggest the need to more closely link these conditions or interventions to preventive health needs and public health goals. The need for action at the community-level and intersection with health equity is also underscored by a recent county-specific study of malnutrition among older Texans (65 +). It found household poverty status, low food access, low educational level, and rurality were all significantly associated with crude death rates from malnutrition [41].

Future federal guidance on/attention to malnutrition, sarcopenia, frailty, and obesity screening, assessment, and interventions in state/territorial plans could cascade down to local OAA agencies’ plans and their interface with public health and health equity initiatives. For example, Massachusetts was identified in our study as having an OAA program multi-year aging plan in the highest quartile of mentions of *nutrition* and with a high number of mentions of *malnutrition/undernutrition/underweight.* A 2021 community nutrition “Be a Nutrition Neighbor” social media campaign in Massachusetts focused on older adult malnutrition as a serious problem and directed individuals to the state’s website that provided a variety of online resources, including those helping address food insecurity [42]. A stronger nutrition stronger focus in OAA program aging plans could also influence opportunities for including nutrition in state Master Plans for Aging” [43]. These Master Plans are often led by state executive/legislative leaders and guide restructuring of state/local policy, programs, and funding to support aging well in the community.

Multiple community-based recommendations and resources are available for favorably impacting the nutrition and health of older adults and could be consulted by federal policymakers. Examples are found in the US *National Blueprint: Achieving Quality Malnutrition Care for Older Adults, 2020 Update* that identified specific recommendations to improve quality of malnutrition care for older adults, including improving quality of malnutrition care practices, improving access, and advancing public health efforts [44]. Global resources that could be leveraged by US federal policymakers include the WHO’s care pathways to manage malnutrition [2]; the Canadian Malnutrition Task Force’s nutrition care pathways, guidance, and recommended screening tools [45]; and a recent United Kingdom impact assessment that documented successful interventions for helping older people maintain a healthy diet and reduce the risk of malnutrition in a community setting [46]. Another important resource is the WHO’s Integrated Care for Older People (ICOPE) implementation framework [47]. ICOPE enables health and long-term care systems/services to respond optimally to the unique, varied, and often complex needs of older people and maximize their intrinsic capacity and functional ability. In addition, ICOPE includes specific recommendations on malnutrition, including that “oral supplemental nutrition with dietary advice should be recommended for older people affected by undernutrition” [48].

In summary, no single policy or program can address the dilemmas facing the US in implementing and monitoring national mandates to improve the nutrition and health of older adults living in the community. Nevertheless, more explicit federal guidance on operational definitions of malnutrition, more complete prevalence estimates for nutrition-related conditions, and strengthening ties to other community health programs may help position OAA nutrition programs to better support ACL’s mission to “maximize the independence, well-being, and health of older adults” [24].

### Strengths and limitations

One strength of our study is that, to our knowledge, it is the first to review US state/territorial OAA program multi-year aging plans and identify mentions of specific nutrition terms, including conditions with potential impact on healthy aging. It also provides a benchmark for evaluating OAA program multi-year aging plans and ACL guidance going forward, as 2020 OAA Reauthorization provisions related to malnutrition become fully implemented. In addition, the results provide information that may be useful for future research on how national nutrition guidance translates to local actions.

Our study had several limitations; it was not a formative program evaluation, but rather a baseline screen of nutrition term mentions in state/territorial OAA program multi-year aging plans. The terms selected were limited to those focused on conditions/interventions related to muscle mass, body composition, and functionality, and did not include all those possibly associated with or influenced by nutrition. Further, the method for identifying defined nutrition terms in aging plans was a simple count and may have been subject to error. Yet when investigators initially independently reviewed a small sample of the same aging plans, differences in counts were limited. The use of the US ADvancing States website was a logical, publicly available resource. More current aging plans may have been available on state/territorial websites, but a search of these 50 + individual websites was beyond the investigation’s scope. Another limitation is that while federal guidance for US state/territorial OAA program multi-year aging plans exists, it is unknown how closely this guidance may be followed by state/territorial aging units which still display wide variability in their OAA program multi-year aging plans.

## Conclusions

Although nationally representative data on malnutrition, sarcopenia, and frailty are currently limited in the US, it is possible and necessary to obtain community prevalence rates of these conditions to help guide community programs like the OAA nutrition programs. More explicit federal guidance is an important public health policy tool for encouraging state/territorial OAA program multi-year aging plans and Title VI grants for Native Americans to include nutrition-related conditions as part of their measurable goals/objectives, strategies/actions, and program outcomes to better support healthy aging.

## Data Availability

The data that support the findings of this study are available from the ADvancing States website, which represents the 56 US state/territorial units on aging and disabilities and long-term services/supports directors; http://www.advancingstates.org/initiatives/aging-policy-and-programs/map-state-plans-aging

## Abbreviations

US: United States
OAA: Older Americans Act
DGA: Dietary Guidelines for Americans
ACL: Administration for Community Living
